# Prednisolone Nanoprecipitation with Dean Instability Microfluidics Mixer

**DOI:** 10.3390/nano14080652

**Published:** 2024-04-09

**Authors:** Yu Ching Wong, Siyu Yang, Weijia Wen

**Affiliations:** 1 Department of Physics, The Hong Kong University of Science and Technology, Clear Water Bay, Hong Kong 999077, China; ycwongak@connect.ust.hk (Y.C.W.); phsidney@ust.hk (S.Y.); 2 Thrust of Advanced Materials, The Hong Kong University of Science and Technology (Guangzhou), Guangzhou 510630, China; 3 HKUST Shenzhen-Hong Kong Collaborative Innovation Research Institute, Futian, Shenzhen 518000, China

**Keywords:** Dean flow, Dean instability, micro-mixing, nanoparticle synthesis, inertial microfluidics, non-linear microfluidics

## Abstract

Dean flow and Dean instability play an important role in inertial microfluidics, with a wide application in mixing and sorting. However, most studies are limited to Dean flow in the microscale. This work first reports the application of Dean instability on organic nanoparticles synthesis at De up to 198. The channel geometry (the tortuous channel) is optimized by simulation, in which the mixing efficiency is considered. With the optimized design, prednisolone nanoparticles are synthesized, and the size of the most abundant prednisolone nanoparticles is down to 100 nm with an increase in the Re and De and smallest size down to 46 nm. This work serves as an ice-breaker to the real application of Dean instability by demonstrating its ability in mixing and nanomaterials like nanoparticle synthesis.

## 1. Introduction

Nanoparticle synthesis has been widely studied and applied in various fields in recent years [1,2,3]. Among all the synthetic methods, microfluidics outperforms the others by providing a microscale mixing [4,5]. There are two types of micro-mixing: active mixing, which requires external energy input, and passive mixing, which completely relies on the hydrodynamics in the microchannel. In general, passive mixing provides more simplicity in system [6,7,8] as active mixing usually requires integration with power sources such as an electromagnetic field [9], acoustic field [10] or thermal actuation [11]. Simplicity in the system does not mean simplicity in the channel, as there is no external power source, and passive mixing heavily depends on the design of the channel for the best mixing efficiency [12,13,14]. However, such complexity of channel geometry can be reduced by increasing the Reynolds number (Re):

(1) $$Re=\frac{\rho UH}{\mu }$$

where *ρ*, *U*, *H* and *μ* are the fluid density, average fluid velocity, channel dimension and dynamic viscosity.

An emerging sub-field, inertial microfluidics, has been a promising candidate for sorting [15,16] and passive mixing in the past two decades [17,18]. In inertial microfluidics, the Re is outside the Stokes’ flow regime and lies in an intermediate range of Re, meaning the inertia term in the Navier–Stokes equations is no longer negligible. There are mainly two types of channel geometries, straight and curved channels in inertial microfluidics [19]. Dean flow occurs in curved channels due to the paraboloid nature of the velocity profile, and results in the generation of two counter-rotating vortexes (Dean vortexes) in the plane perpendicular to the main flow direction. Such vortexes promote mixing and the strength can be quantified by the Dean number (De):

(2) $$De=Re\sqrt{\frac{H}{2R}}$$

where *R* is the radius of curvature. There are numerical [20,21] and experimental reports [22,23] on using Dean flow for mixing purposes. Regarding nanoparticle synthesis, X. Yu et al. [24] performed ZIP-8 nanoparticles synthesis using the Dean flow and resulted in nanoparticles from 40 nm to 700 nm. M. Thiele et al. [25] fabricated the gold nanocubes with the edge length down to 53 nm by Dean flow in the zigzag channel.

According to Dean [26] and Reid [27], Dean instability results as the De exceeds a critical value, resulting in the generation of an extra pair of vortexes at the outer wall due to the increase in radial pressure gradient. The four vortexes condition further enhances mixing. There are only a few numerical [28] and experimental studies on the microscale. Nivedita et al. [29] first visualized the secondary flow pattern of low aspect ratio channels in different aspect ratios but only demonstrated the application in particle sorting. Y.C. Wong et al. [30] illustrated the generation of Dean instability in a high aspect ratio channel, proposed a tortuous design, and demonstrated the ability to mix with Dean instability.

This paper presents one of the first few applications of Dean instability in microfluidics mixing. It is an experimental study on the synthesis of nanoparticles using Dean instability. It is a continuing work on a previous study, optimizing the novel tortuous channel design and carrying on to applications. The tortuous channel design was designed specifically for the creation of Dean instability at a lower Re environment compared to other typical channel patterns due to the perturbation of smaller radii [30]. The selection of prednisolone as the application for this study is based on its practical use as a glucocorticoid drug. Previous research has also examined this drug in microchannels [31]. The lack of chemical reaction involved in its precipitation enables a clear focus on the alteration of Re and De in this study.

## 2. Materials and Methods

### 2.1. Fabrication

The devices were fabricated in two phases (image of the process flow shown in Supplementary Materials). In the first phases, the silicon mould for polydimethylsiloxane (PDMS) was fabricated at The Hong Kong University of Science and Technology Nanosystem Fabrication Facility (HKUST NFF) using standard lithography and deep reactive ion etching (DRIE). The positive photoresist (HPR 506) was spun, exposed in Karl Suss MA6 (Munich, Germany) and developed by the FHD5 developer. The etching depth across the silicon wafer was 90 μm ± 2 μm measured by Tencor P-10 Surface Profiler (Hwaseong-si, Republic of Korea). The second stage was PDMS device fabrication. Here, 10:1 PDMS of 50 g was poured onto the mould and cured in the oven at 80 °C (24 h). The glass slide (SLItech, Lagos, Nigeria, microscope slides, MS-13) was bonded to the PDMS after 3 min of plasma treatment in the plasma cleaner (Harrick, Pleasantville, NY, USA, PDC-001-HP) followed by baking at 80 °C (40 min). The connecting tube is Tygon Microbore Autoanalysis Tubing, Anyang, Republic of Korea.

### 2.2. Sample Preparation

The prednisolone was purchased from Sigma-Aldrich (St. Louis, MO, USA) and the absolute ethanol was purchased from Scharlau (Barcelona, Spain). The saturated prednisolone solution was prepared by putting the excess amount of prednisolone into absolute ethanol. The addition of prednisolone was stopped only if oversaturation was observed after shaking of 10 mins (Fisher Vortex Genie 2, Hampton, NH, USA). The oversaturated solution was then centrifuged at 14,000 rpm for 5 min (Sigma 3-18K, Tokyo, Japan) and the saturated prednisolone was removed and ready for use. The prednisolone solution was later used to mix with deionized (DI) water in the microchannel.

### 2.3. Experimental Setup and Data Analysis

**Figure 1:**
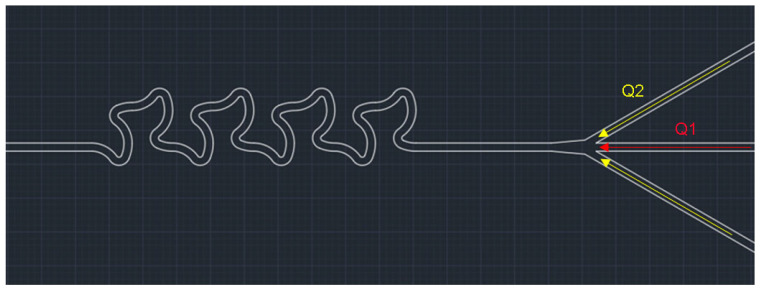
Optimized channel with mixing length of 2L, Q1: DI water, Q2: prednisolone solution.

The channel geometry is shown in Figure 1, it consists of three radii of curvatures (270 μm, 50 μm and 200 μm) in a single unit with 60 μm in width and 90 μm in height. This geometry was chosen after optimization by simulation. There are two channel lengths: L (3227 μm) and 2L (6454 μm). The infusion was carried out by PHD 2000 Infusion (Harvard Apparatus, Cambridge, MA, USA), and the total flow rates (Q1 + Q2) were 175 μL/min (Re = 39), 250 μL/min (Re = 56), 525 μL/min (Re = 117), 700 μL/min (Re = 156), 875 μL/min (Re = 194) and 1050 μL/min (Re = 233) while the ratio of Q2:Q1 is always 1:2.5 to maintain the solvent to DI water ratio. The experiment was carried out under the light microscope (Olympus IX71, Tokyo, Japan) to ensure no blockages of precipitate occurred during the solution collection, an image of the setup is attached in Supplementary Materials. The collected solution was then washed and dried on diced silicon wafers on the hotplate at 40 °C until all liquid evaporated. The particles were then observed under the scanning electron microscope (SEM) (JSM-7100F, Tokyo, Japan). The SEM images were analyzed in ImageJ 1.53 t to obtain the size distribution, and the graphs were plotted in Origin 2018. And the most abundant particles’ sizes and percentage in amount are discussed.

### 2.4. Simulation

The simulation was performed with COMSOL Multiphysics 6.0, and the pattern was imported from AutoCAD 2021. Laminar flow and the transport of diluted species modules were used and water was considered in the simulation. The size of meshing elements was chosen to range from 1.64 μm to 8.71 μm after we evaluated the convergence of data, and the results of the meshing convergence test are attached in Supplementary Materials. The Navier–Stokes equation and the continuity equation for incompressible Newtonian fluid solved in the laminar flow module were

(3) $$\rho \frac{Du}{Dt}=-\nabla p+\mu \nabla^{2}u+F$$

(4) $$\frac{\partial p}{\partial t}+\nabla \cdot \left(\rho u\right)=0$$

where u is the velocity field, *p* is the pressure and F is any external force. The boundary condition of the walls was no slip and the pressure at the outlet was set to be zero.

The velocity field obtained from the laminar flow module was then coupled with the transport of the diluted species module. The initial concentration in the channel was set to be 0 mol/m^3^ and two species of 1 mol/m^3^ and 0 mol/m^3^ were injected from the inlets for mixing. The convection–diffusion equation responsible is given as

(5) $$\frac{\partial c}{\partial t}=D\nabla^{2}c-u\cdot \nabla c$$

where *c* is the concentration.

We obtained the velocity profiles and concentration profiles for analysis. The simulation mainly served as an optimization of channel parameters purposes with assistance in flow visualization. The concentration graphs were analyzed and compared by the Mixing Index (*MI*):

(6) $$MI=1-\frac{\sqrt{\frac{1}{N}\Sigma_{i}^{N}{\left(I_{i}-\bar{I}\right)}^{2}}/\bar{I}}{\sqrt{\frac{1}{N}\Sigma_{i}^{N}{\left(I_{oi}-\bar{I_{o}}\right)}^{2}}/\bar{I_{o}}}$$

where $I_{i}$ and $\bar{I}$ are the local concentration magnitude and the average concentration at the cross-section of the outlet, $I_{oi}$ and $\bar{I_{o}}$ are the local concentration magnitude and the average concentration at the cross-section of the inlet, and *N* is the amount of data.

## 3. Results and Discussion

### 3.1. Optimization of the Channel Geometry by Simulation

Microfluidics mixer enhances mixing by stretching and folding the fluid elements. Such a feature exponentially increases the interfacial area between two species and allows layers of fluid to interact by diffusion or convection. In the condition of Dean instability, the strength of vortexes (value of De) is a major factor in facilitating convection and hence mixing. From the equation of De, the characteristic dimension and radius of curvature are related to the geometrical design. The characteristic dimension in this study is already the smallest value considering the strength of plasma bonding [32]. As a result, only the radius of curvature is optimized and its variation refers to the change in magnitude of the centripetal force (F∝1/R).

The channel geometries are optimized by changing the radius of the second and third curvature with a fixed first radius of curvature. The range of radius in the second and third turns are from 40 μm to 80 μm and from 160 μm to 270 μm, respectively, with an interval of 10 μm, and two repeating units are studied in each case. Simulations were carried out and the MI were calculated and compared. The MI of the top 10 patterns in mixing at Dean instability is shown in Table 1.

The second radius of curvature plays a dominant role in the mixing, as the De there is significantly larger than other parts of the repeating unit. Ideally, the smaller the better for the radius and the result reflects that the second radius of curvature in the best mixing pattern is 50 μm, the second smallest value in the range. For the third radius of curvature, the result of 200 μm, which is close to the middle value of the range, also meets our expectations. As we previously studied, the tortuous design behaves slightly differently in the flow due to the rolled-up velocity profile [30]. The centripetal force and pressure gradient “cooperate” for vortex formation. The third radius of curvature acts for reinforcement purposes and, hence, a moderate value is appropriate.

**Figure 2:**
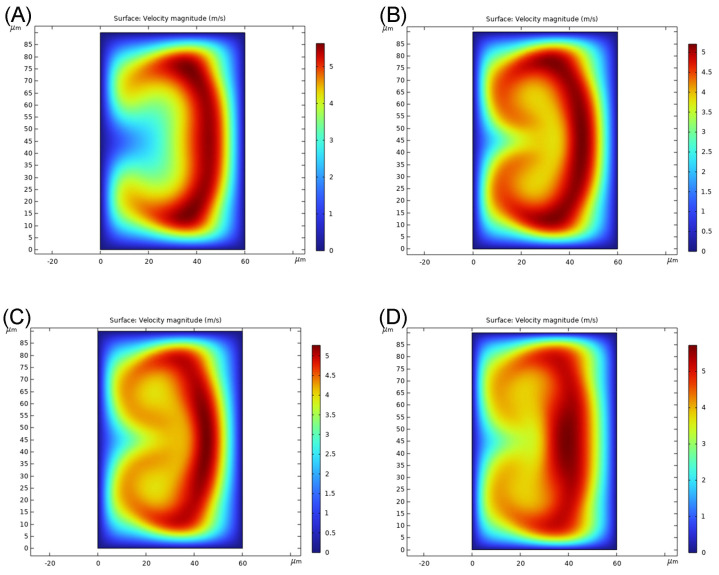
Velocity profile at the end of the third radius of curvature (Re = 233). (**A**) (De = 156) 2nd radius of curvature: 80 μm, 3rd radius of curvature: 200 μm. (**B**) (De = 198) 2nd radius of curvature: 50 μm, 3rd radius of curvature: 200 μm. (**C**) (De = 221) 2nd radius of curvature: 40 μm, 3rd radius of curvature: 200 μm. (**D**) (De = 221) 2nd radius of curvature: 40 μm, 3rd radius of curvature: 160 μm.

There is a piece of additional information the optimization result reveals. It is the ratio of force for the second curvature to the third curvature. Since the tortuous channel presents a “cooperation” behaviour, it is reasonable to assume there is an optimized ratio. From Table 1, the top few mixing patterns that perform the best all have a ratio close to 4. However, the ratio itself does not complete the picture; we need to consider the velocity profile. Due to the design nature, the second radius of curvature acts as a fillet to connect the channel and the angle of curvature changes with the radius. Such difference in angle affects the swirl [33] and hence the development of flow. As shown in Figure 2, at a longer second radius of curvature of 80 μm (Figure 2A), the fluid elements of the highest velocity are pushed toward the side and elongate along the wall. At a shorter second radius of curvature of 50 μm (Figure 2B), the velocity profile looks similar but with more roll-up features. For the second radius of curvature in 40 μm (Figure 2C), it is obvious that the fastest fluid elements do not elongate along the side, meaning a non-ideal velocity profile. We further present the velocity profile with 40 μm as the second radius of curvature in the top 10 mixing patterns (Figure 2C) and it is shown that the loss in the rolled-up velocity profile is more obvious due to the difference in the third radius of curvature. As a result, we think besides the higher De the better the mixing principle, the presence of a rolled-up velocity profile and an appropriate ratio in radii also needs to be considered for the best mixing performance in the tortuous design at Dean instability condition.

In addition, the mixing length (data shown in Supplementary Materials) is affected as the circumference changes with the radius. However, we do not observe a major effect of the mixing length at Dean instability condition. It may due to the near-complete mixing condition (MI close to 1). The effect of the mixing length is further explored in the experiment.

### 3.2. Prednisolone Nanoparticles Synthesis at Mixing Length L

In general, the drug nanoparticles are synthesized by either “top-down” or “bottom-up” approaches. The top-down approach is the breaking of larger particles while the bottom-up approach is the building up of particles from molecules in solution. The synthesis of nanoparticles in microfluidics adapts the bottom-up approach by mixing the solvent with anti-solvent. This means the mechanism is in the molecular range.

**Figure 3:**
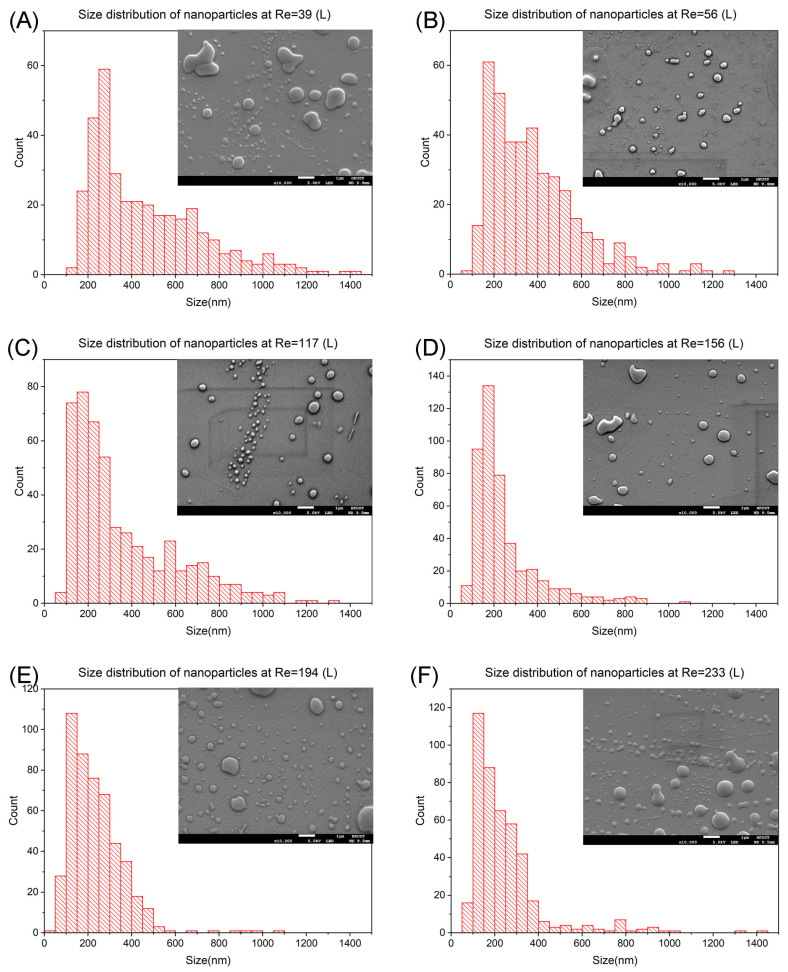
Size distribution and SEM images of prednisolone nanoparticles at mixing length L. (**A**) SEM image and size distribution at Re = 39, De = 33. (**B**) SEM image and size distribution at Re = 56, De = 48. (**C**) SEM image and size distribution at Re = 117, De = 99. (**D**) SEM image and size distribution at Re = 156, De = 132. (**E**) SEM image and size distribution at Re = 194, De = 165. (**F**) SEM image and size distribution at Re = 233, De = 198.

Figure 3 presents the size distribution charts of prednisolone particles and a corresponding SEM image to each Re ranging from 39 to 223 (De ranging from 33 to 198). The effect of increases in Re and De is discussed. Figure 3A shows the most abundant nanoparticles at Re = 39 are in 250 nm to 300 nm, occupying 16.9% of the total. As the Re increases to 56, the most abundant size range changes to 150 nm to 200 nm with 15.5% (Figure 3B). The most abundant size range remains at 150 nm to 200 nm when the Re increases to 117 and 156 (Figure 3C,D). Moreover, the percentage increases to 15.9% and 29.4%. The size range decreases to 100 nm to 150 nm when the Re increases to 194 and 233 (Figure 3E,F). The percentages of the most abundant nanoparticles are 22.1% and 26.5%, respectively, in these two Re.

The result agrees with our hypothesis. For Dean flow regime, there is only one pair of Dean vortexes. As the Re increases from 39 (De = 33) to 56 (De = 48). The transverse velocity and the strength of Dean vortexes increase. This enhances the mixing, leading to a drop in the most abundant particle size from 250 nm to 300 nm to 150 nm to 200 nm. It remains as Dean flow as Re = 117 and 156 (De = 99 and 132). The strengthening of Dean vortexes in this transition is not strong enough for a reduction in particle size, but there is an increase in the percentage of the most abundant particle for 13.9%.

The flow condition changes from Dean flow to Dean instability as the Re proceeds to 194 (De = 165). The value of De matches our previous work as we observed Dean instability at De = 172 in the unoptimized channel. The most abundant particle size further decreases to 100 nm to 150 nm. It is because the mixing is further improved as the pressure-gradient generated vortexes arise. Similar to the previous transition, there is not a significant change in flow behaviour when Re changes to 233 (De = 198), resulting in no change in particle size but an increase in the percentage of 1.8%. From this set of data in mixing length L, we confirm the nanoparticle size drops with an increment in Re and De.

### 3.3. Prednisolone Nanoparticles Synthesis at Mixing Length 2L

The prednisolone nanoparticles synthesized in 2L are analyzed similarly along with the size distribution graphs and SEM images. We would like to observe if the mixing length has no significant effect as the simulation shows, with a larger difference by doubling the length.

**Figure 4:**
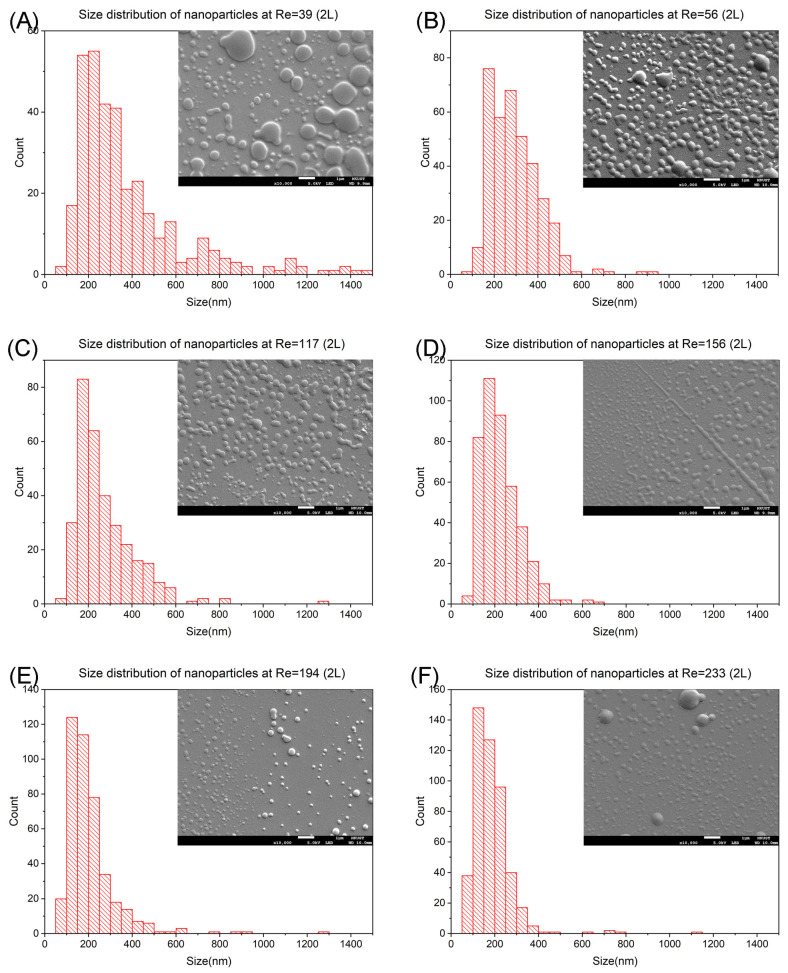
Size distribution and SEM images of prednisolone nanoparticles at mixing length 2L. (**A**) SEM image and size distribution at Re = 39, De = 33. (**B**) SEM image and size distribution at Re = 56, De = 48. (**C**) SEM image and size distribution at Re = 117, De = 99. (**D**) SEM image and size distribution at Re = 156, De = 132. (**E**) SEM image and size distribution at Re = 194, De = 165. (**F**) SEM image and size distribution at Re = 233, De = 198.

In Figure 4A, the most abundant nanoparticles at Re = 39 are in 200 nm to 250 nm with 16.0%. The size range changes to 150 nm to 200 nm with 20.8% (Figure 4B) at Re = 56. The nanoparticles size range is the same as Re increases to 117 and 156 (Figure 4C,D). And the corresponding percentages are 25.7% and 26.2%. The size range decreases to 100 nm to 150 nm when the Re increases to 194 and 233 (Figure 4E,F) with 29.2% and 31.0%.

The overall change in the size of the nanoparticles and the percentage are similar as in the case of mixing length L. The fluid in Dean flow first gains more momentum, reducing the particle size down to 150 nm to 200 nm, then transiting to Dean instability, further reducing the particle size to 100 nm to 150 nm. And the most abundant particle size remains the same in some transitions, but all with an increment in the percentage of abundance in those phases (+5.4% in Dean flow and +1.7% in Dean instability).

There is one major difference between L and 2L: the drop in particle size at the lowest flow rate (from 250 nm to 300 nm down to 200 nm to 250 nm). It is due to the incomplete mixing at the lowest flow rate. For the other flow rates, the most abundant particles sizes remain the same because the mixing is near completion, hence with no change in size. However, we do observe some effect of the change in mixing length, which is not observed from the simulation. That is the amount of the most abundant particle increases with mixing length. We believe there is a higher probability of the particles with the highest percentage in size to be synthesized and the increase in mixing length reinforces such behaviour. This leads to an overall increase in the percentage number. As a result, we think the mixing length is crucial when there is no complete mixing. And for a complete mixing case, only the purity of nanoparticles is improved.

## 4. Conclusions

The first experimental study of Dean instability in real application at the microscale is reported. The tortuous channel is optimized numerically with the consideration of the radii of curvature, showing the importance of radii of curvature and a rolled-up velocity profile. We concluded the nanoparticles size decreases as the Dean flow transits to Dean instability, and the mixing length has a limited effect in the Dean instability regime. This work shows the promising mixing ability of the tortuous channel. And it demonstrates a huge potential in other applications involving micro-mixing apart from nanotechnology like nanoparticles synthesis.

## Figures and Tables

**Table 1:** Top 10 best-performing mixing patterns (two repeating units).

2nd Radius of Curvature [μm]	3rd Radius of Curvature [μm]	Mixing Index (cor. to 3 sig. fig.)
50	200	0.988
60	260	0.987
60	210	0.986
50	190	0.986
60	190	0.985
40	160	0.985
70	200	0.981
70	220	0.981
60	170	0.980
70	230	0.979

## Data Availability

The data used in this study are available from the corresponding author upon reasonable request.

## Supplementary Materials

The following supporting information can be downloaded at: https://www.mdpi.com/article/10.3390/nano14080652/s1, Figure S1: work flow of the fabrication; Figure S2: Experimental setup; Figure S3: Convergence of velocity; Table S1: Meshing tested for the convergence test; Table S2: Top 10 best performed mixing patterns (two repeating units) at Dean instability.
