# Supplementary material for: Prednisolone Nanoprecipitation with Dean Instability Microfluidics Mixer
Source: Nanomaterials (Basel). 2024 Apr 9;14(8):652. doi: 10.3390/nano14080652 (PMC11054107; doi:10.3390/nano14080652)
Supplement: Supplementary file 1 [file nanomaterials-14-00652-s001.zip › nanomaterials-2942936-supplementary.pdf]

Supplementary Materials

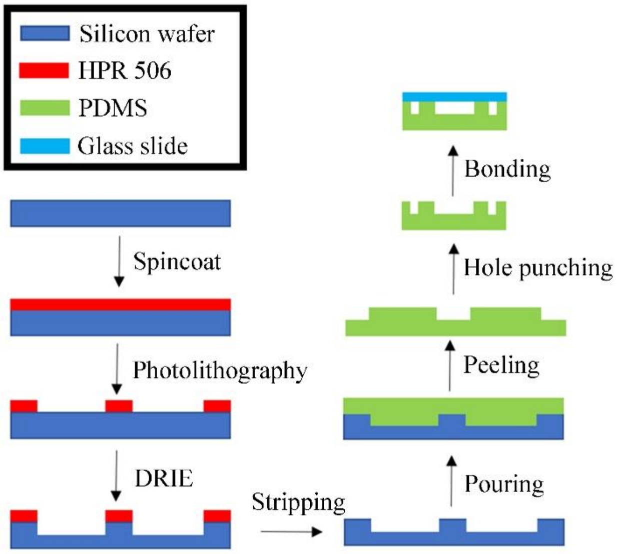

Supp. Figure S1 work flow of the fabrication

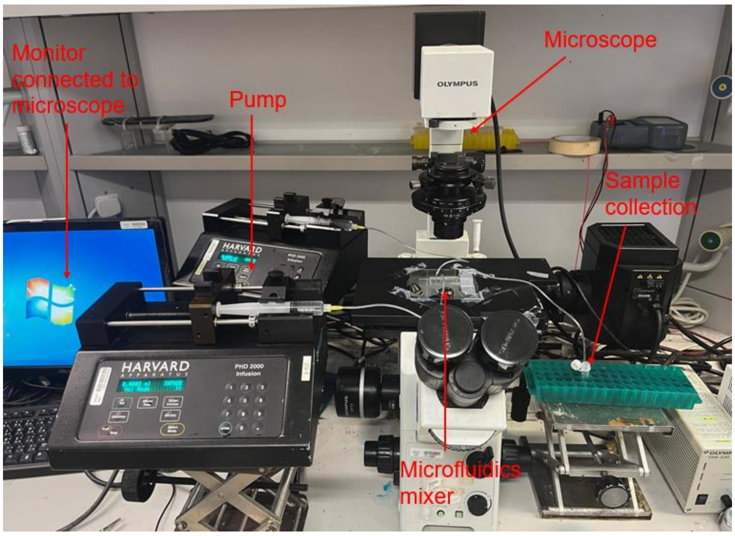

Supp. Figure S2 Experimental setup

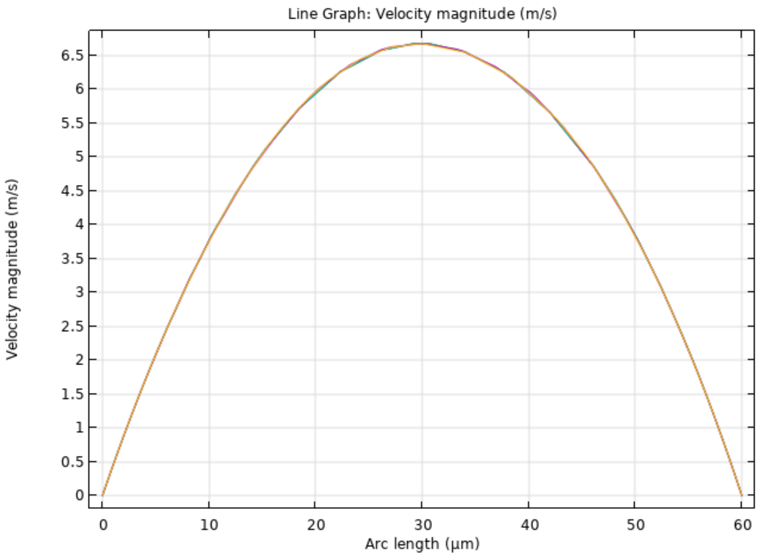

Supp. Figure S3 Convergence of velocity

The velocity profile along the horizontal midline near the inlet is investigated. The velocity profile converges to a parabolic shape as the meshing changes from 1 to 6.

Supp. Table S1 Meshing tested for the convergence test

	Meshing1	Meshing2	Meshing3	Meshing4	Meshing5	Meshing6
Maximum element size[μm]	54.2	32.9	21.4	16.4	11	8.71
Minimum element size [μm]	11.5	8.22	6.57	4.93	3.29	1.64
Maximum element growth rate	1.4	1.3	1.25	1.2	1.15	1.13
Curvature factor	1	0.9	0.8	0.7	0.6	0.5
Resolution of narrow regions	0.3	0.4	0.5	0.6	0.7	0.8

Supp. Table S2 Top 10 best performed mixing patterns (two repeating units) at Dean instability

2nd radius of curvature[μm]	3rd radius of curvature[μm]	Mixing length	Mixing Index (cor. to 3 sig. fig)
50	200	403	0.988
60	260	362	0.987
60	210	384	0.986
50	190	409	0.986
60	190	395	0.985
40	160	451	0.985
70	200	373	0.981
70	220	366	0.981
60	170	408	0.980
70	230	360	0.979

As shown in the Supp. Table S2, the mixing length varies in the top 10 of channel geometries at Dean instability. The trend does not follow the longer mixing length better mixing efficiency principle which is usually applicable in the traditional microfluidics mixing. An additional possible reason here may be the difference in the mixing length is too subtle to notice the result. From the experimental result in the main text, it is observed that the mixing length does matter as in the lowest flow rate we tested. This proves the insufficient difference in mixing length for the optimization simulation.
